# In Vitro Activity of Neem (*Azadirachta indica*) Oil on Growth and Ochratoxin A Production by *Aspergillus carbonarius* Isolates

**DOI:** 10.3390/toxins11100579

**Published:** 2019-10-05

**Authors:** Mariana Paiva Rodrigues, Andrea Luciana Astoreca, Águida Aparecida de Oliveira, Lauranne Alves Salvato, Gabriela Lago Biscoto, Luiz Antonio Moura Keller, Carlos Alberto da Rocha Rosa, Lilia Renée Cavaglieri, Maria Isabel de Azevedo, Kelly Moura Keller

**Affiliations:** 1 Programa de Pós-Graduação em Ciência Animal, Escola de Veterinária, Universidade Federal de Minas Gerais, Belo Horizonte, Minas Gerais 31270-901, Brazil; 2 Centro de Investigación y Desarrollo en Fermentaciones Industriales, Consejo Nacional de Investigaciones Científicas y Técnicas, Facultad de Ciencias Exactas, Universidad Nacional de La Plata, La Plata, Buenos Aires B1900ASH, Argentina; 3 Departamento de Microbiologia e Imunologia Veterinária, Instituto de Veterinária, Universidade Federal Rural do Rio de Janeiro, Seropédica, Rio de Janeiro 23890-000, Brazil; 4 Departamento de Zootecnia e Desenvolvimento Agrossocioambiental Sustentável, Faculdade de Veterinária, Universidade Federal Fluminense, Niterói, Rio de Janeiro 24230-340, Brazil; 5 Consejo Nacional de Investigaciones Científicas y Técnicas, Departamento de Microbiología e Inmunología, Facultad de Ciencias Exactas, Físico Químicas y Naturales, Universidad Nacional de Río Cuarto, Río Cuarto, Córdoba X5804BYA, Argentina; 6 Departamento de Medicina Veterinária Preventiva, Escola de Veterinária, Universidade Federal de Minas Gerais, Belo Horizonte, Minas Gerais 31270-901, Brazil

**Keywords:** mycotoxins, essential oils, ecophysiology

## Abstract

*Aspergillus carbonarius* is a saprobic filamentous fungus, food spoiling fungus and a producer of ochratoxin A (OTA) mycotoxin. In this study, the in vitro antifungal activity of neem oil (0.12% p/p of azadirachtin) was evaluated against the growth of six strains of *A. carbonarius* and the production of OTA. Four different concentrations of neem oil were tested in addition to three incubation times. Only the concentration of 0.3% of neem oil inhibited more than 95% of the strain’s growth (97.6% ± 0.5%), while the use of 0.5% and 1.0% of neem oil showed lower antifungal activity, 40.2% ± 3.1 and 64.7% ± 1.1, respectively. There was a complete inhibition of OTA production with 0.1% and 0.3% neem oil in the four strains isolated in the laboratory from grapes. The present study shows that neem essential oil can be further evaluated as an auxiliary method for the reduction of mycelial growth and OTA production.

## 1. Introduction

Members of the *Aspergillus* spp., among many other toxigenic fungi, have been found to have a strong ecological link with human food supplies [1]. They are often associated with food and animal feed during drying and storage but may also occur as plant pathogens. Black aspergilli, *Aspergillus* classified into the section *Nigri* [2], have been isolated from a wide variety of food and are distributed worldwide (animal feed, cereals, cocoa, coffee, dried fruits, fruits, garlic, olives, onions) and are considered as common fungi causing food spoilage and biodeterioration of other materials [3,4]. Furthermore, they are important producers of ochratoxin A (OTA), the main species involved in OTA biosynthesis is *Aspergillus carbonarius*, commonly isolated from tropical regions as a contaminant of vineyards [5].

OTA can result in toxic effects to human and animal species. This toxicity may be acute or chronic, and varies depending on the amount of OTA absorbed, the exposure time, species affected, age and sex [6]. Among the toxic effects it is possible to highlight nephrotoxicity (tubular necrosis), hepatotoxicity, teratogenicity, enteritis and carcinogenesis [7]. OTA is also classified as Group 2B, possibly carcinogenic to humans, according to the International Agency for Research on Cancer [8]. OTA has also been correlated to Balkan endemic nephropathy (BEN) [9]. Due to all the economic, human and animal health damages that the contamination of *A. carbonarius* and OTA can cause, the prevention and control of these fungi and mycotoxin are of extreme importance.

Essential oils (EO) are a complex mixture of volatile, odoriferous, aromatic compounds that have antioxidant and antimicrobial components [10,11]. In addition, studies have already shown that EOs improve the flavor and palatability of feed, thus increasing voluntary feed intake by animals [12,13]. This could make these substances good for biological control against fungi and mycotoxins. The physical nature of essential oils (i.e., low molecular weight combined with pronounced lipophilic tendencies) allow them to penetrate the cell membrane more quickly than other substances [14]. Moreover, several studies have focused on the possible use of different essential oils as biological drivers against aflatoxigenic fungi [15,16,17,18].

Neem oil is an EO extracted from different parts of the neem tree *(Azadirachta indica)*, a native tree from the drier regions of Asia and Africa that is considered a very important medicinal plant. So far, more than 300 phytochemicals, chemically diverse and structurally complex, have been extracted and isolated from different parts of this tree [19]: from leaves—azadirachtin (AZ), nimonol, nimocinol and nimocinolide; from barks—gallic acid, gallocatechin and epicatechin; from seeds—azadirachtin (AZ), azadiradione, nimbin, salannin and epoxyazadiradione [20,21,22,23,24]. These chemical compounds have demonstrated a wide range of unusual effects against a wide spectrum of pests (insects, fungi, and viruses) [25]. Neem EO is commonly used as an antipyretic, natural insecticide, antimicrobial, antimalarial agent, antibacterial, antifungal, antiviral and for the treatment of leptospirosis [26,27,28,29,30]. Neem leaf extract (NLE) also has anti-fertility effects, by NLE–induced oocyte apoptosis [31]. Even though they are effective against a wide spectrum of insects, fungi and viruses, these compounds have low toxicity to mammals [25], which reveals the great potential of this oil for use a possible biological control of fungi and mycotoxins. There are no values of reference for the use of neem oils and extracts but based on the “lead compound concept” the European Commission, Health and Consumers Directorate-General established 0.1 mg/kg body weight/day as the acceptable daily intake (ADI) for the lead compound AZ [32]. 

Thus, the aims of the present work were to evaluate: (i) the efficacy of different concentration levels of neem oil on growth parameters: lag phase and growth rate of six ochratoxigenic *Aspergillus carbonarius* strains; (ii) the potential to control ochratoxin A production by these strains grown on Czapek yeast extract agar (CYA) at different incubation times.

## 2. Results

The effects of different concentrations of neem oil on the percentage of growth inhibition of six *Aspergillus carbonarius* strains assayed on a CYA medium are shown in Table 1.

**Figure 1:**
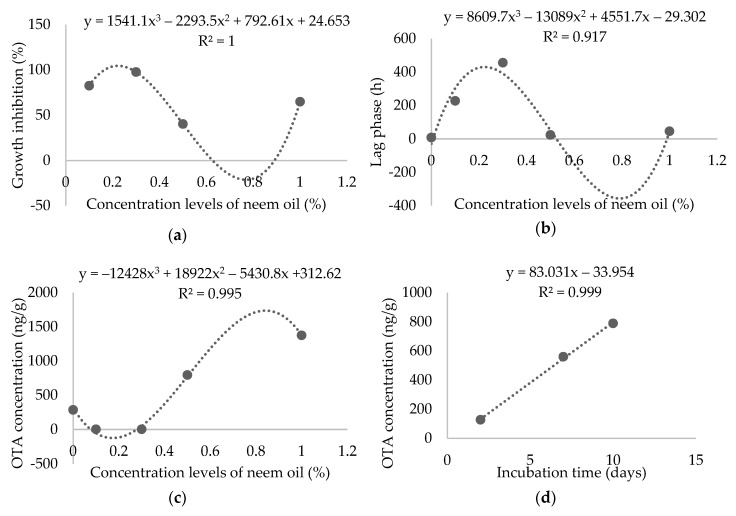
(**a**) The cubic polynomial fitting curve for percentage of growth inhibition of *A. carbonarius* strains against different concentrations of neem oil; (**b**) fitted regression curve using non-linear cubic splines for lag phase vs. different concentrations of neem oil; (**c**) the fitted cubic spline model to the OTA concentration data; (**d**) the linear curve fitting of incubation time vs. OTA concentration.

Among the four concentrations of neem oil screened, 0.1% and 0.3% inhibited more than 82% and 97%, respectively, of the growth of *A. carbonarius* strains, which indicate a high antifungal activity (>80%). The 0.5% concentration had a poor anti-fungal effect (<50%), whereas the application of 1.0% of neem oil had a medium effect (59–71%). Although the regression analysis indicated significant linear dose-responses (Table 2), the data fit a more cubic polynomial model (Figure 1). 

Mean lag phase (h) of six *A. carbonarius* strains at different concentration levels of neem oil are shown in Table 3. 

Neem oil concentrations of 0.3% and 0.1% had a significant effect on lag phase, increasing the time needed for each strain to reach the exponential phase. The regression analysis showed a significant polynomial trend model correlation of different neem oil concentrations with the lag phase (Table 4), and the cubic trend seemed to better fit the model (Figure 1). 

The effect of neem oil treatments on OTA production by six *A. carbonarius* strains assayed after 2, 7 and 10 days of incubation is shown in Table 5. 

There was a complete inhibition in OTA production with the addition of 0.1% and 0.3% of neem oil for the four strains isolated from grapes whereas the two reference strains assayed (FRR5690 and A2034) produced low levels of OTA (28.2 and 22.2 ng/g, respectively) at 10 days of incubation. The absence of OTA production was also observed at two days of incubation and 1% of neem oil for FRR5690 and RCG4 strains.

The overall treatment time showed an increase in OTA production as incubation time increased and the regression analysis indicated significant linear dose-responses (Table 6; Figure 1). 

An increase in OTA production was observed at 0.5% and 1% of neem oil. These two concentrations stimulated the OTA production at the end of the incubation period in 116.8 ± 78.8% and 498.8 ± 385.4%, respectively.

Single factors (concentration of neem oil and incubation time) as well as two-way interaction had a significant effect on OTA production by *A. carbonarius* strains studied (*p* < 0.001) (Table 6).

## 3. Discussion

The effect of natural or synthetic compounds on *Aspergillus* section *Flavi* species growth and aflatoxin production has already been described by some authors. Gowda, Malathi and Suganthi [33] studied the effect of some chemical and herbal compounds on the growth of other toxicogenic specie, *Aspergillus parasiticus*, and it was observed that neem oil at 0.5% had moderate anti-fungal activity (84% reduction vs. control), and at 0.2% and 0.1% a low antifungal activity, 52% and 36%, respectively. A lower percentage of reduction in fungal biomass (51%) was obtained by Zeringue and Bhatnagar [34] who studied the effects of neem leaf volatiles on submerged cultures of the same species. The contradictions between these results and the present study can possibly be explained by the different biochemical pathways that regulate the synthesis of the different mycotoxins produced by studied *Aspergillus* species and by the differences in the composition and therefore, the properties of each oil fraction. Razzaghi-Abyaneh et al. [35] agreed with those authors previously mentioned since they reported that neem leaf and seed extract can cause morphological alterations in the exposed mycelia, and then lead to cellular destruction. 

Sitara et al. [36] concluded that the ideal concentrations for the reduction of the *Alternaria alternata* growth was 0.1% and 0.15% of neem oil extracted from seeds. These results corroborate the ideal concentrations of 0.1% and 0.3% found in this study. On the other hand, Bhatnagar and McCormick [37], studied the effects of the neem leaf extracts at 1%, 5%, 10%, 20% and 50% (*v/v*) on growth of *A. parasiticus* and concluded that it had no significant alterations at the mycelial growth. These results were very similar to Zeringue and Bhatnagar [38], that evaluated the effects of neem leaf extract on *Aspergillus flavus* and found only 4–7% of growth reduction.

On the other hand, Zeringue, Shih and Bhatnagar [39] studied the effects of clarified neem oil on growth in submerged and plated cultures of aflatoxigenic *Aspergillus* spp. which resulted in an increase of 11–31% measured by mycelia mass. Garcia and Garcia [40] agreed with those authors previously mentioned since they reported that neem did not inhibit either growth or aflatoxin production by *A. flavus* and *A. parasiticus*.

The 0.1% and 0.3% concentrations of neem oil completely inhibited the production of OTA for the four strains isolated from grapes. This can be explained since none of the four wild strains at 0.1% and three strains (RCG1, RCG2 and RCG3) at 0.3% had reached the exponential growth phase. However, at 0.5% and 1.0% concentrations, all the strains, except RCG1, showed increased production of OTA; this possibly occurred because these concentrations inhibited less of the mycelial growth in all of the six strains assayed. Another possibility is that the presence of the neem oil and the AZ compound in high concentrations could lead to an exacerbated oxidative stress situation by the fungus and an increase in OTA production.

According to the previous data in literature [41] the sensitivity of *Aspergillus* spp. to oxidative status perturbations is closely related to the production of mycotoxins. Several publications [41,42] addressed the exact mechanisms included in regulating the development and secondary metabolism of many *Aspergillus* spp. The production of mycotoxins is triggered by oxidative stress; an increase in reactive oxygen species (ROS) levels can increase mycotoxins levels, noting this phenomenon as one of the defense mechanisms of fungal cells. The tolerance of *A. flavus* and *A. parasiticus* isolates to oxidative stress has also been shown to be correlated with their levels of aflatoxin production. Roze et al. [43] showed that conidia of isolates with higher levels of aflatoxin production also exhibited greater viability when cultured in ROS-amended medium.

Finally, another possibility is that higher concentrations (0.5% and 1.0%) of neem oil could have exceeded the solubility limits of the tested medium and the effective compounds did not have the same activity as in the lower concentrations [44,45]. 

These results are divergent to Bhatnagar and McCormick [37] who found that using 10% concentration of neem leaf extract reduces 98% of *A. parasiticus* aflatoxins production even if there is no inhibition of the mycelial growth. Allameh et al. [46] concluded that the concentration needed to reduce 90% of the aflatoxin production from *A. parasiticus* was 50% of neem leaf extract (*v/v*). This concentration was 150 times greater than the ideal concentration of neem oil found in this study. Razzaghi-Abyaneh et al. [35] also found a high reduction (91.3%) of aflatoxin production per µg of mycelia by *A. parasiticus* using 1.56% of neem extracts from seeds and leaf.

While many compounds and substances have been found to effectively inhibit fungal growth and aflatoxin production, others have stimulatory properties and affect the biosynthesis or bioregulation of aflatoxins, just like what happened with the utilization of 0.5% and 1.0% concentration of neem oil [47]. Nowadays, the information about action mechanisms of these compounds on *Aspergillus* species is limited, however it is possible to assure that the neem oil has important antifungal properties against *A. carbonarius*.

## 4. Conclusions

These findings clearly indicate the use of neem oil in low concentrations, such as 0.1% and 0.3%, is a good possibility as an auxiliary control method for mycelial growth reduction in *Aspergillus carbonarius* strains and the inhibition of ochratoxin A production. Mycotoxin contamination in food poses serious health hazards to animals and humans. Very few scattered reports are available on the effects of plant oils on growth of ochratoxigenic fungi. This study can contribute to the knowledge to develop effective anti-mycotoxigenic natural products for reduction of mycotoxigenic fungi and mycotoxins in foods.

## 5. Materials and Methods 

### 5.1. Fungal Strains

Six *Aspergillus carbonarius* strains were evaluated as follows: two reference strains (FRR5690 and A2034) from the CSIRO Collection Centre, Australia and four *A. carbonarius* strains (RCG1, RCG2, RCG3 and RCG4) isolated from dry grapes in Argentina [48]; all of them were OTA producers on yeast extract sucrose (YES, HiMedia Laboratories Pvt. Ltd., Mumbai, India) medium (2% yeast extract, 15% sucrose). 

### 5.2. Culture Medium

Commercial neem oil (Base Fértil Agrícola, Cravinhos, SP, Brazil) was used in this study. According to the manufacturer’s certificate of analysis, neem oil was extracted from seeds and contained 0.12% p/p of azadirachtin (= 1200 ppm). Neem oil was added to the Czapek yeast extract agar (CYA, HiMedia Laboratories Pvt. Ltd., Mumbai, India) at final concentrations of 0.1%, 0.3%, 0.5% or 1.0% (*v/v*) at 45 °C. Plates containing CYA media without neem oil were used as control.

### 5.3. Inoculation and Incubation Conditions 

Spore suspensions of the six *A. carbonarius* strains were obtained by scraping the surface of a 7-day-old colony cultured in 2% malt extract agar (MEA, HiMedia Laboratories Pvt. Ltd., Mumbai, India) and transferring the conidia to a tube containing 10 mL of sterile distilled water supplemented with 0.1% Tween 20. The solution was homogenized and read in a spectrophotometer (530 nm) to obtain a transmittance between 80% and 82%, which corresponds to 1–5 × 10^6^ colony forming units per milliliter (CFU/mL). Then the Petri plates were needle-inoculated centrally with 10 µL of the spore suspension. The plates were incubated at 25 °C ± 2 for a maximum of two weeks. 

### 5.4. Growth Assessment 

Two perpendicular diameters of the growing colonies were measured daily until the colony reached the edge of the plate or for a maximum of two weeks. The percentage inhibition of diameter growth (PIDG) values were determined according to the equation as below:

$$\mathrm{PIDG}\;\left(\%\right)\;=\;\frac{\mathrm{Diameter}\;\mathrm{of}\;\mathrm{sample}\;-\;\mathrm{Diameter}\;\mathrm{of}\;\mathrm{control}\;}{\mathrm{Diameter}\;\mathrm{of}\;\mathrm{control}}\;\times \;100$$

Growth rate (mm day^−1^) was calculated by linear regression of colony diameter against time for each strain at each set of conditions tested, and the time at which the line intercepted the x-axis was used to calculate the lag phase (h) in relation to isolate and essential oil. In all cases, the experiments were carried out with three replicates per treatment. The growth of fungal cultures containing different concentrations of neem oil was compared with that of the control culture that was grown with no EO.

### 5.5. Ochratoxin A Extraction from Culture

Ochratoxin A production was analyzed after 2, 7 and 10 days of incubation. The methodology proposed by Bragulat, Abarca and Cabañes [49] with some modifications was used. On each sampling occasion, three agar plugs were removed from different points of the colony and extracted with 1 mL of methanol. The mixture was centrifuged at 14,000 rpm for 10 min. The solutions were filtered (syringe filters, 17 mm, 0.45 µm, nylon membranes), evaporated to dryness, and re-dissolved in 200 µL of mobile phase (acetonitrile:water:acetic acid, 57:41:2) and the extracts injected into the high-performance liquid chromatography system.

### 5.6. OTA Detection and Quantification

The OTA production was detected and quantified by reversed phase in a high performance liquid chromatography system Hewlett Packard Serie 1100 (HP/Agilent, Santa Clara, CA, USA) with fluorescence detection (λ_exc_ 330 nm; λ_em_ 460 nm) using a C_18_ column (Supelcosil^™^ LC-ABZ, 150 × 4.6 mm, 5 µm particle size), connected to a precolumn (Supelguard^™^ LC-ABZ, 20 × 4.6 mm, 5 µm particle size). The mobile phase was pumped at 1.0 mL/min. The injection volume was 100 µL and the retention time was around 4 ± 1 min. The detection limit of the analyses was 1 ng/g [50]. 

### 5.7. Statistical Analyses

Statistical analyses were conducted using PROC GLM in SAS program (SAS Institute Inc., Cary, NC, USA). The differences between growth inhibition percentage, lag phase and OTA production at different concentration levels of neem oil by six *Aspergillus carbonarius* strains at 2, 7 and 10 days were analyzed statistically by analyses of variance (ANOVA). The statistical models used in ANOVA considered the effects of the dependent variables and “strains” as a covariate within the different concentration levels of neem oil. The independence of the covariate was formally checked. Means were compared by Fisher’s LSD test to determine the influence of the neem oil on the ecophysiology of the strains assayed [51]. Orthogonal polynomial contrasts were used to determine linear, quadratic and cubic responses to neem oil. The OTA production data contained some results of “not detected” (ND). That is, the OTA concentration was not detected above the detection limit (DL) of the used method. The actual concentration represented by ND is some value below the DL, however, the analytical method cannot determine whether the ND is truly zero or some unquantifiable value between zero and the DL. For this situation we used the substitution method, replacing the ND with the DL value (1 ng/g). In the cases of “not growth” (NG) results, we performed the substitution with zero. 

## Figures and Tables

**Table 1:** Percentage of growth inhibition of six *A. carbonarius* strains produced by different concentrations of neem oil on a Czapek yeast extract agar (CYA) medium.

Strains	Concentration Levels of Neem Oil (%)			
	0.1	0.3	0.5	1.0
FRR5690	73.7 ± 8.5	96.3 ± 0.3	45.1 ± 1.7	58.5 ± 1.7
A2034	91.1 ± 1.1	98.8 ± 0	34.5 ± 2.8	70.8 ± 3.1
RCG1	97.8 ± 2.2	100 ± 0	47.3 ± 9.3	62.2 ± 4.0
RCG2	87.1 ± 0.6	98.9 ± 0	36.4 ± 4.4	65.6 ± 4.9
RCG3	96.3 ± 2.3	98.2 ± 0.6	40.9 ± 1.1	61.5 ± 3.6
RCG4	49.2 ± 4.5	93.7 ± 1.3	37.1 ± 1.7	69.5 ± 2.9
Means ± SD	82.5 ± 17.7 ^b^	97.6 ± 2.2 ^a^	40.2 ± 6.1 ^d^	64.7 ± 5.4 ^c^

SD: standard deviation. ^a–d^ Means with different letters are significantly different (*p* < 0.001).

**Table 2:** Outputs of the ANOVA with single-degree-of-freedom orthogonal polynomial contrasts for the effects of different concentrations (C) of neem oil on growth inhibition of six *A. carbonarius* strains (ST).

Source	df	Type III SS	MS	F	*p*-Value
(C)	3	32,923.07	10,974.36	118.79	<0.0001*
Covariate: (ST)	1	340.49	340.49	3.69	0.0591
Error	67	6189.78	92.38		
Contrast	df	Contrast SS	MS	F	*p*-value
(C)-linear	1	11,077.80	11,077.80	119.91	<0.0001*
(C)-quadratic	1	392.93	392.93	4.25	0.0431*
(C)-cubic	1	21,452.34	21,452.34	232.21	<0.0001*
Parameter			Estimate	SE	*p*-value
(C)-linear			−110.94	10.13	<0.0001*
(C)-quadratic			9.34	4.53	0.0431*
(C)-cubic			154.39	10.13	<0.0001*

“Strains” was not a significant covariate. Overall model R^2^ = 0.84. df: degrees of freedom; SS: sum of squares; MS: mean squares; F: Fisher–Snedecor test. * Significant *p* < 0.001.

**Table 3:** Lag phase (h) of six *A. carbonarius* strains at five different concentration levels of neem oil.

Strains	Concentration Levels of Neem Oil (%)				
	0 (Control)	0.1	0.3	0.5	1.0
FRR5690	7.4 ± 3.8	101.7 ± 27.0	≥540	29.6 ± 1.3	52.5 ± 2.6
A2034	5.6 ± 1.7	53.2 ± 8.2	≥540	16.6 ± 4.2	40.7 ± 4.5
RCG1	13.1 ± 0.8	531.7 ± 14.4	≥540	31.9 ± 6.2	40.5 ± 6.7
RCG2	8.4 ± 2.0	87.6 ± 4.1	≥540	26.5 ± 2.0	45.8 ± 3.3
RCG3	2.9 ± 2.6	≥540	≥540	18.0 ± 3.7	41.3 ± 2.8
RCG4	6.5 ± 0.5	154.0 ± 15.4	36.0 ± 6.9	15.7 ± 3.6	47.9 ± 0.6
Means ± SD	7.5 ± 3.7 ^c^	226.6 ± 207.5 ^b^	456.2 ± 193.3 ^a^	23.2 ± 7.4 ^c^	44.9 ± 5.6 ^c^

SD: standard deviation. ^a–c^ Means with different letters are significantly different (*p* < 0.001).

**Table 4:** Outputs of the ANOVA with single-degree-of-freedom orthogonal polynomial contrasts for the effects of different concentrations (C) of neem oil on lag phase of six *A. carbonarius* strains (ST).

Source	df	Type III SS	MS	F	*p*-Value
(C)	4	2,640,845.40	660,211.35	42.09	<0.0001*
Covariate: (ST)	1	23,815.89	23,815.89	1.52	0.2213
Error	83	1,301,845.28	15,684.88		
Contrast	df	Contrast SS	MS	F	*p*-value
(C)-linear	1	29,536.64	29,536.64	1.88	0.1737
(C)-quadratic	1	1,431,427.76	1,431,427.76	91.26	<0.0001*
(C)-cubic	1	347,184.26	347,184.26	22.13	<0.0001*
Parameter			Estimate	SE	*p*-value
(C)-linear			−128.48	93.62	0.1737
(C)-quadratic			−1057.38	110.68	<0.0001*
(C)-cubic			444.36	94.45	<0.0001*

“Strains” was not a significant covariate. Overall model R^2^ = 0.67. df: degrees of freedom; SS: sum of squares; MS: mean squares; F: Fisher–Snedecor test. * Significant *p* < 0.001.

**Table 5:** Ochratoxin A (OTA) concentration (ng/g) produced by six *A. carbonarius* strains at five different concentration levels of neem oil at the incubation times assayed.

Strains	Incubation Time (Days)	OTA Concentration (ng/g)				
		0	0.1	0.3	0.5	1.0
FRR5690	2	62.5 ± 4.7	Nd	Nd	368.8 ± 34.3	Nd
	7	267.9 ± 46.5	Nd	Nd	637.7 ± 35.4	2817.2 ± 219.9
	10	334.3 ± 7.4	Nd	28.2 ± 3.2	986.3 ± 86.1	2954.7 ± 54.4
A2034	2	117.9 ± 2.1	Nd	Nd	468.2 ± 12.1	110.5 ± 4.0
	7	225.5 ± 10.0	13.8 ± 0.0	Nd	624.4 ± 18.4	454.5 ± 4.5
	10	325.7 ± 20.3	22.2 ± 2.2	Nd	741.3 ± 21.6	720.2 ± 1.3
RCG1	2	201.6 ± 2.2	Nd	NG	232.2 ± 25.9	168.0 ± 12.2
	7	278.8 ± 10.8	Nd	NG	358.0 ± 8.0	268.3 ± 0.6
	10	396.3 ± 7.8	Nd	NG	432.9 ± 2.0	258.5 ± 26.5
	2	102.6 ± 6.4	Nd	Nd	512.7 ± 30.7	160.2 ± 11.0
RCG2	7	573.2 ± 19.2	Nd	Nd	2281.2 ± 79.2	1808.3 ± 50.4
	10	758.6 ± 58.6	Nd	Nd	1785.0 ± 10.2	6164.4 ± 329.4
	2	227.8 ± 49.8	Nd	Nd	232.5 ± 56.6	280.1 ± 81.4
RCG3	7	384.3 ± 25.8	Nd	Nd	2232.6 ± 273.4	1904.2 ± 155.8
	10	528.6 ± 33.0	Nd	Nd	1403.5 ± 300.7	3054.8 ± 199.9
	2	86.4 ± 7.5	Nd	Nd	542.9 ± 41.1	Nd
RCG4	7	160.7 ± 6.1	Nd	Nd	183.5 ± 5.5	1507.4 ± 9.4
	10	203.8 ± 6.8	Nd	Nd	278.5 ± 16.5	2103.7 ± 102.9
	2	122.3 ± 68.8 ^cC^	Nd ^dC^	0.8 ± 0.4 ^dC^	392.9 ± 132.8 ^bC^	120.1 ± 105.1 ^aC^
Means ± SD	7	309.6 ± 142.2 ^cB^	3.13 ± 4.9 ^dB^	0.8 ± 0.4 ^dB^	1025.2 ± 869.7^bB^	1460.0 ± 905.3 ^aB^
	10	424.6 ± 184.7 ^cA^	4.5 ± 8.2 ^dA^	5.4 ± 10.6 ^dA^	965.7 ± 597.7 ^bA^	2542.7 ± 1987.7 ^aA^

SD: standard deviation; NG: not growth; Nd: not detected (limit of detection 1ng/g). ^a–d^ Means with different lowercase letters in the row are significantly different (*p* < 0.001). ^A–C^ Means with different capital letters in column are significantly different (*p* < 0.001).

**Table 6:** Outputs of the ANOVA with single-degree-of-freedom orthogonal polynomial contrasts for the effects of different concentrations (C) of neem oil on ochratoxin A (OTA) production of six *A. carbonarius* strains (ST) at three incubation times (T).

Source	df	Type III SS	MS	F	*p*-Value
(C)	4	75,146,241.75	18,786,560.44	47.27	<0.0001*
(T)	2	20,290,698.84	10,145,349.42	25.52	<0.0001*
Covariate: (ST)	1	569,135.62	569,135.62	1.43	0.2326
(C)* (T)	8	37,954,380.17	4,744,297.52	11.94	<0.0001*
Error	254	100,956,999.01			
Contrast	df	Contrast SS	MS	F	*p*-value
(C)-linear	1	47,609,459.27	47,609,459.27	119.78	<0.0001*
(C)-quadratic	1	24,443,018.97	24,443,018.97	61.50	<0.0001*
(C)-cubic	1	1,320,779.85	1,320,779.85	3.32	0.0695
Parameter			Estimate	SE	*p*-value
(C)-linear			2969.27	271.30	<0.0001*
(C)-quadratic			2517.36	321.01	<0.0001*
(C)-cubic			−494.56	271.30	0.0695
Contrast	df	Contrast SS	MS	F	*p*-value
(T)-linear	1	19,669,576.14	19,669,576.14	49.49	<0.0001*
(T)-quadratic	1	621,122.70	621,122.70	1.56	0.2124
Parameter			Estimate	SE	*p*-value
(T)-linear			661.14	93.98	<0.0001*
(T)-quadratic			−203.49	162.78	0.2124

“Strains” was not a significant covariate. Overall model R^2^ = 0.57. df: degrees of freedom; SS: sum of squares; MS: mean squares; F: Fisher–Snedecor test. * Significant *p* < 0.001.
